# Tumor microenvironment in ovarian cancer peritoneal metastasis

**DOI:** 10.1186/s12935-023-02854-5

**Published:** 2023-01-25

**Authors:** Shuangshuang Mei, Xing Chen, Kai Wang, Yuxin Chen

**Affiliations:** 1 Taizhou Hospital of Zhejiang Province Affiliated to Wenzhou Medical University, Xi Men Road, Taizhou, 317000 Zhejiang China; 2 Taizhou Hospital of Zhejiang Province Affiliated to Wenzhou Medical University (Enze Hospital, Taizhou Enze Medical Center Group), Tong Yang Road, Taizhou, 318053 Zhejiang China

**Keywords:** Ovarian cancer, Tumor microenvironment, Metastasis

## Abstract

Ovarian cancer (OC) is one of the most common gynecological malignancies with high morbidity and mortality. The peritoneum is one of the most common metastatic sites in ovarian cancer, involving large amounts of ascites. However, its mechanism is unclear. The peritoneal microenvironment composed of peritoneal effusion and peritoneum creates favorable conditions for ovarian cancer progression and metastasis. Here, we reviewed the peritoneal metastasis patterns and molecular mechanisms of ovarian cancer, as well as major components of the peritoneal microenvironment, peritoneal effusion, and immune microenvironment, and investigated the relationship between the peritoneal microenvironment and ovarian cancer metastasis.

## Highlights

1. Ovarian cancer (OC) is one of the most common gynecological tumors. It commonly develops peritoneal metastasis and is associated with poor prognosis. However, the molecular biology and pathophysiology of ovarian cancer are not known.
2. Emerging evidence highlight the role of tumor microenvironment as a crucial factor in driving peritoneal metastatic progression.

## Background

Ovarian cancer (OC) is a significant cause of female cancer mortality and morbidity, being the second new diagnosed case and the first leading death in gynecologic cancers [1]. Epithelial ovarian cancer (EOC) is the most common pathological type of OC, accounting for about 90% [2]. Despite having diverse pathological types, peritoneal metastasis is a common feature in patients with EOC and signals dismal [3], contributing to malignant ascites accumulation composed by the complex tumor-promoting microenvironmental network in the peritoneal cavity [4]. Although the peritoneal cavity provides a stage for OC peritoneal metastasis, numerous challenges of intra-abdominal metastasis in OC remain presented due to the underlying mechanism is not clearly defined, involving recurrence and morbidity. Exploring the mechanisms of intraperitoneal metastasis of ovarian cancer could improve molecular diagnosis and treatment.

The tumor microenvironment (TME) within the peritoneal cavity is a major component determining peritoneal metastasis in OC [5]. The OC TME consists of diverse cell types including tumor cells and host stromal cells, blood vessels, and the extracellular matrix (ECM) such as collagen [6]. While TME of OC intra-abdominal metastasis including malignant ascites and peritoneal metastasis is poorly understood, studies indicate that the cooperation of tumor and stromal cells support the dissemination of OC cancer cells within the peritoneal cavity [7], as the main reason for the poor prognosis and adverse outcomes [8, 9]. Clinically, OC patients with metastasis within the peritoneal cavity are related to poor prognosis [10]. Here, we review how OC formulate peritoneal metastasis and how the TME within the peritoneal cavity supports the OC peritoneal metastasis.

## Models of OC in the colonization of the peritoneal cavity

**Fig. 1:**
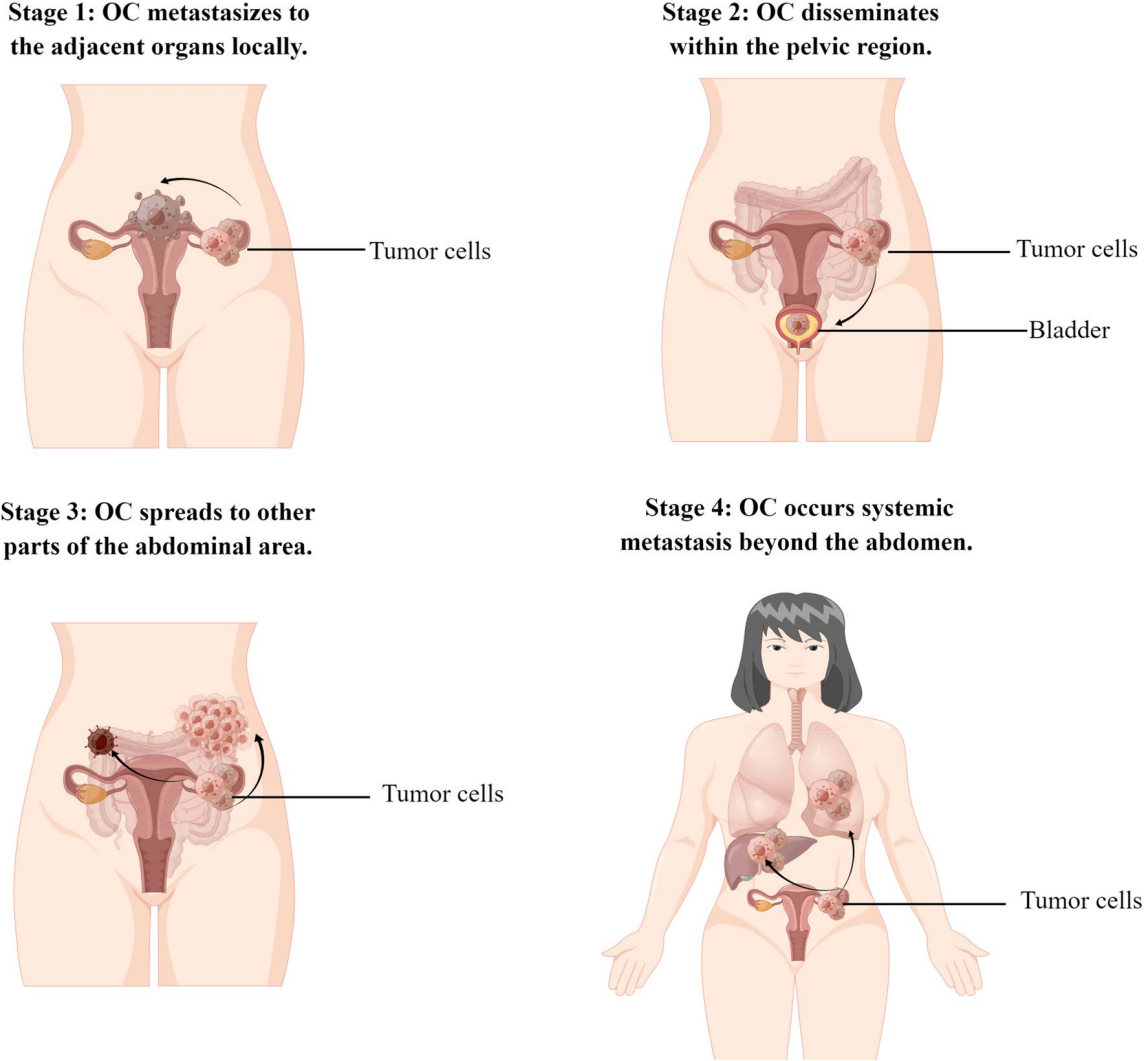
Different stages of ovarian cancer

Lymphatic metastasis usually occurs in pelvic and para-aortic lymph nodes. Hematogenous spread is the least common metastatic path [11]. Currently, it is well accepted that OC metastasis proceeds through a series of stages (Fig. 1). Consistent with most tumors, the common initial stage of OC metastasis spreads to the adjacent organs such as the surrounding connective tissues, fallopian tubes, and uterus [12]. Then, the OC disseminates within the pelvic and abdominal area through transcoelomic, hematogenous, and lymphatic routes [4]. Systemic metastasis including liver and lung is relatively less frequent and usually appears in the late-stage of OC progression [7, 13].

**Fig. 2:**
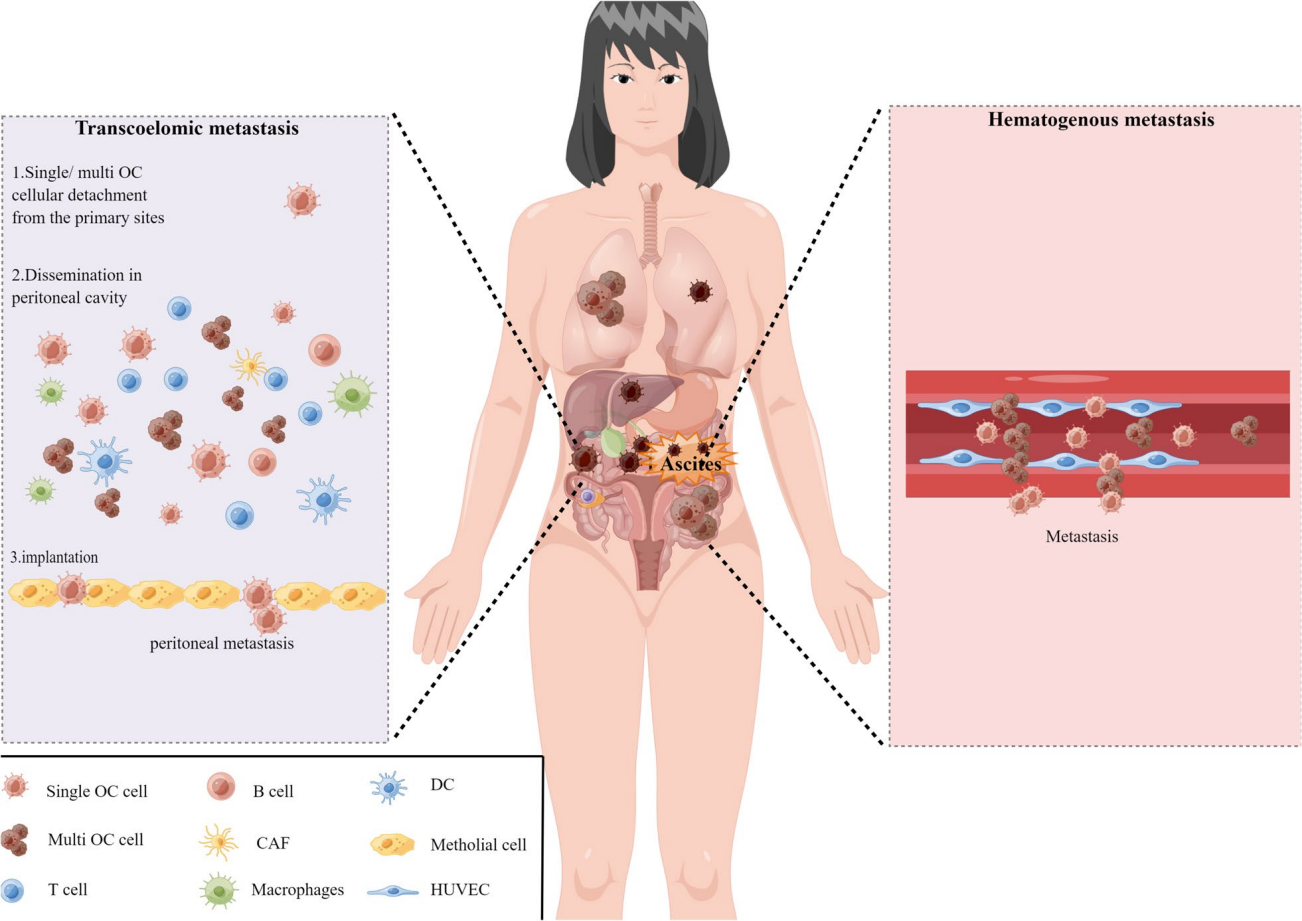
Two main pathways of peritoneal metastasis in ovarian cancer

Compared to other tumors, ovarian cancer metastasis occurs most frequently in the omentum or peritoneum. Studies reported that almost 70% of patients with ovarian cancer presented peritoneal cavity metastasis at the time of diagnosis [4, 14]. As shown in Fig. 2, OC tumor cells are preferentially home to the omentum and develop metastatic lesions, while micro-metastases are often presented on the peritoneal surfaces. Immunohistochemical analysis of metastatic omentum and peritoneal tissues shows poor microvasculature perfusion [15]. In addition, the metastatic omentum and peritoneal tissues lose the normal collagen network.

## The hypothesis of the OC peritoneal metastasis model

Currently, two hypotheses have been proposed for the peritoneal metastasis model in ovarian cancer. The first hypothesis, related to the “seed and soil” hypothesis [16] is that the peritoneal metastasis of OC originates from circulating tumor cells within the peritoneal cavity, which preferentially metastasize to the peritoneum through transcoelomic, hematogenous, or lymphatic route. The second hypothesis, referred to as the metaplasia hypothesis, is that the OC metastatic omental sites are the synchronized malignant transformation of the peritoneum or omentum due to the similar lineage between ovarian epithelium and omentum [4]. Although the “seed and soil” hypothesis has been commonly accepted historically, some studies have indicated that de-novo malignant transformation occurs in multiple lesions of the OC peritoneal metastasis [17]. However, the “seed and soil” hypothesis or the “metaplasia hypothesis” cannot completely clarify the full picture of OC peritoneal metastasis, and small studies currently explore this issue. Thus, how to form peritoneal metastasis needs further study.

### Transcoelomic metastatic route of EOC

There are several reasons why OC tumor cells typically metastasize to the abdominal peritoneum or omentum through the transcoelomic pathway. First, the ovaries are close to the abdominal cavity in terms of anatomical location. Besides, there is no peritoneal coverage of the ovary, resulting in no physical barrier separating the tumor from the abdominal cavity. Therefore, the location and features of the ovaries provided an ideal place for transcoelomic metastasis in the peritoneal cavity. Second, given the common linage between the omental surface epithelium and the ovarian epithelium [4], the omentum and peritoneum surfaces provide the soil of tumor-supportive microenvironment for OC metastasis according to the seed-and-soil hypothesis [16]. Lastly, the larger area of the cavity and the existence of peritoneal fluid assist the metastasis of OC tumor cells in the peritoneal cavity.

Detachment from the primary tumor, dissemination within the peritoneal cavity, and implantation in the peritoneum of OC tumor cells are significant in successful transcoelomic metastasis. This is a complex process that requires the adaptation of OC tumor cells within the circulating ascites (Fig. 2).

### Step 1: peeled from the primary tumor

The initial step of the transcoelomic journey is the relative passive exfoliation of EOC tumor cells from the primary sites into the peritoneal cavity, which could be further promoted by the peritoneal fluids [4, 18, 19]. Although the underlying mechanism of tumor cell detachment from its primary lesion is not well defined, it is commonly accepted that the loss of cell–cell contact involved in the disruption of OC cell adhesion contributes to the process. Integrins are transmembrane glycoproteins that can regulate cell functions such as adhesion, invasion, and survival [20, 21], involved in the intraperitoneal metastatic cascade of OC. Aberrant expression and functions of integrins impair cell adhesion between cells. For instance, cleaving α3-integrin contributes to the detachment of OC cells from the primary ovarian mass [22]. Up-regulation of α-integrin promotes OC metastasis by assisting OC cells to attach more efficiently [23]. Another potential factor related to the detachment of OC cells is E-cadherin. Downregulation of E-cadherin regulated by Snail promoted the initial tumor cell detachment and migration during metastatic progression in OC [24]. Another controversial event in OC metastasis is an epithelial-to-mesenchymal transition (EMT). Unlike other epithelial cancer, the role of EMT in OC progression is unclear. Similar to other epithelial tumors, some investigators consider that detachment of OC cells from the primary mass related to peritoneal metastasis experiences EMT [25]. However, others argue that separation of OC cells from the primary sites involved in peritoneal metastasis without undergoing EMT, as the metastatic tumor cells still express E-cadherin [26]. Ovarian epithelial cells occur EMT process in response to culture rather than acquire a real invasive phenotype [24, 27]. Thus, most of the EMT phenomenon in OC has only been revealed in vitro.

### Step 2: dissemination in the peritoneal cavity

Following cell department, ovarian cancer cells could survive and disseminate within the peritoneal cavity. Malignant ascites provides a metastatic milieu for OC. On the one hand, the mechanical forces and natural flow resulting from the physiological movement of ascites provide aspects for the intraperitoneal implantation metastasis of the detached OC cells [28]. On the other hand, ascites facilitate OC transcolomic metastasis by generating a unique TME consisting of tumor cells, stromal cells, and an acellular compartment [29]. The ascites microenvironment supports the dissemination and seeding of OC cells within the peritoneal cavity [7]. The detailed role of ascites in OC metastasis is specified in Part III (Peritoneal Microenvironment and OC peritoneal metastasis). Although malignant ascites is crucial for OC intraperitoneal metastasis, detached cancer cells survive in ascites after overcoming many obstacles such as spheroid formation, anoikis resistance, immunological surveillance, and so on.

Aggregation is a hallmark of the metastatic OC cells within the peritoneal cavity. Tumor cells of ascites could be present as separate cells or aggregate cells such as spheroids, facilitating implantation on the surface of metastatic organs. Aggregated ovarian tumor cells survive and disseminate within the peritoneal cavity through increased complement resistance due to the ability of antibodies and complement to penetrate the OC spheroids becoming insufficient [30]. Interaction between integrin α5β1 and fibronectin promotes ovarian cancer cell aggregation in vitro [31]. UBR5 promotes the spheroid formation of OC cells through regulating p53 protein expression [32]. In addition, the ascitic tumor cells could format spheroids containing stromal cells or immune cells, which contribute to peritoneal dissemination [33]. For example, upregulating integrin α5 in ascitic tumor cells facilitates spheroids formation between tumors and fibroblasts, involved in early peritoneal metastasis [34]. However, the precise mechanisms of the role of spheroid formation related to OC peritoneal metastasis remain unclear.

Anoikis refers to a specific process of cellular apoptosis when cells lose cell–matrix interactions. Several studies have revealed that after leaving the primary lesions, OC cells could survive within the peritoneal cavity by overcoming anoikis [35]. Targeting anoikis of OC cells remises OC metastasis [36–38]. Several molecules including RAB25 small GTPase [39], integrin members [40, 41], LRRC15 [38] regulate the anoikis resistance of OC cells. Furthermore, resistance to anoikis promotes OC metastasis by activating yes-associated protein 1 (YAP1) pathway [42]. Thus, anoikis is a hallmark of normal endothelial cells while OC cells develop resistance to anoikis during dissemination within the peritoneal cavity.

Immune escape is another hallmark of the metastatic OC cells within the peritoneal cavity. High levels of immunomodulators such as IL-2, TNF exist in OC malignant ascites [43]. Besides, OC cells in ascites induce apoptosis of CD95 positive immune cells through exosomal CD95 ligand [44], impairing normal immune response. OC cells also inhibiting proliferation of immune cells through releasing metabolites [45, 46]. Ascites could also recruit regular T(Treg) cells and inhibit specific anti-tumor immunity, promoting tumor progression [47]. Malignant ascites fluid impairs metabolism and normal function of T cells through perturbing glucose uptake, which leads to immune evasion in OC TME [48]. Thus, the detached OC cells presenting as single cells or spheroids survive within the intraperitoneal cavity by overcoming many obstacles and metastasize further.

### Step 3: implantation

Similar to the process of OC cells leaving from the primary sites, the underlying mechanisms involving the peritoneal metastases establishment of circulating OC cells are undefined, but seem to be associated with the dynamic interaction between the OC cells and mesothelium. It is accepted that transcoelmic metastasis is likely the predominant pathway of OC spread, while distant metastasis via hematogenous route is less important. The process includes adhesion of the metastatic OC cells to mesothelial cells, infiltration of the metastatic OC cells to the sub-mesothelial matrix and contributes to peritoneal implantation finally.

The metastatic OC cells within the peritoneal cavity adhere to the first barrier of the peritoneum which consisted of human peritoneal mesothelial cells (HPMCs), damaging the mesothelium and infiltrating into the sub-mesenchymal matrix and destructing the peritoneal barrier. Aberrant molecular expression of OC cells is related to the attachment of detached tumor cells to peritoneal mesothelium. Cancer antigen 125 (CA125), a glycoprotein, is overexpressed on OC cells and promotes adhesion and erodibility of OC cells into the peritoneal mesothelial layer through binding to mesothelin (MSLN) [49, 50]. Additionally, matrix-degrading enzymes including matrix metalloproteinases (MMP) are vital to OC metastasis to peritoneum because the basement membrane of peritoneal tissues is rich in collagen and fibronectin. Over-expression of MMP2 on the disseminated OC cells facilitates connecting to peritoneal mesothelium through cleaving fibronectin and vitronectin [51]. Upregulation of MMP14 on the OC cellular surface facilitates tumor metastasis through degrading collagen IV of the basement membrane and fibrillar collagens of the sub-mesothelial stromal matrix [22, 52]. Besides, chemokine CXCL12-CCR4 mediates the attachment of the OC cells to peritoneal mesothelial cells [53]. In addition, the disseminated OC cells attach to the peritoneal mesothelium by regulating hyaluronan expression on mesothelial cells. Angiogenesis is another significant process in metastatic lesions because tumor growth needs neo-blood vessel formation to supply enough nutrients. Stromal cells in the peritoneum supports the OC cell growth through regulating angiogenesis related molecules. Malignant ascites also facilitates the implantation of OC cells in the peritoneum. The mechanical force resulting from elevated intra-peritoneal pressure assists OC cells adhere to the peritoneum and promoting metastasis [28]. Besides, malignant ascites promotes the mesothelial-to-mesenchymal transition of mesothelial cells located at the peritoneal surface, which provides the opportunity for OC cells to erode the sub-mesothelial matrix [54, 55]. Then, metastatic OC cells that disrupt the peritoneal barrier preferentially invade specific structures of the peritoneum such as the “milky spots” in the omentum, initiating an accelerated invasive growth process. The metastatic OC cells interact with mesenchymal, immune, and endothelial cells of the peritoneal tumor microenvironment in a specific peritoneal structure such as “milky spots” to form a pro-tumor microenvironment. OC is easily transferred to the papillary structure of the omentum, which consists mainly of immune cells such as macrophages, lymphocytes, and natural killer cells [10, 56, 57]. According to the “seed and soil” theory, “milky spots” is suitable soil for ovarian cancer cells to survive and grow. Thirdly, the metastatic OC cells infiltrating into the specific peritoneal structures such as “milky spots” facilitate peritoneal interstitial fibrosis and destroy peritoneal structures. Lastly, TME of the metastatic lesions formats neovascularization and supports the metastatic tumor cells’ survival and growth. Angiogenesis is another important process in metastatic lesions, as tumor growth requires the formation of new blood vessels to provide adequate nutrition. Interstitial and tumor cells in the peritoneum support OC cell growth by regulating angiogenesis-associated molecules.

Based on the above studies, we speculate that the dynamic crosstalk between peritoneal stromal cells and disseminated OC cells is vital to fostering the complicated metastatic process. Thus, a better understanding of the OC metastatic tumor microenvironment within the intra-peritoneal cavity will aid in elucidating tumor progression.

### Hematogenous metastatic route of EOC

Recently, a growing number of studies have shown that the hematogenous pathway also plays important roles in tumor metastasis of OC [58, 59], which is initiated by lympho-vascular space invasion. Clinically, inferior vena cava filters increase the risk of hematogenous spread in OC by inducing platelet activation and proinflammatory reaction [60]. Besides, circulating tumor cells detached from the primary lesions could be detected in OC patients at the time of early diagnosis [61–63]. The molecular mechanisms of hematogenous metastasis in OC are also complex. In this process, the metastatic tumor cells need to penetrate the endothelial surface and enter the circulation. Upregulation of genes such as LUM in the OC primary sites is more prone to hematogenous metastasis through promoting extracellular matrix deposition [64]. Down-regulation of CCR4 inhibits hematogenous metastasis by suppressing CTCs of OC cells [65]. Besides, OC cells can also metastasize to the omentum through the hematogenous route via ErbB3/NRG1 axis [59]. Therefore, although hematogenous route in OC metastases is relatively rare, this uncommon pathway should not be ignored, requiring further exploration.

## Peritoneal microenvironment and OC peritoneal metastasis

According to the theory of “seed soil”, the peritoneal tumor microenvironment is composed of diverse cell types including peritoneal mesothelial cells (PMs), fibroblasts, adipocytes, immune cells, endothelial cells, mesenchymal stem cells, etc. and extracellular matrix provides a suitable soil for the formation of peritoneal metastasis in OC. Interaction between tumor cells and the TME can be mainly mediated through direct physical contact, soluble molecules released by the paracrine pathway, bioactive molecules transmission by exosomes, and malignant peritoneal effusion.

## The role of main cell types within the peritoneal cavity in OC peritoneal metastasis

The OC TME consisting of both ascites in a liquid-state and metastatic niches in the solid-state microenvironment is complex. OC displays easy metastasis to the omentum, which is a large visceral peritoneum, located between the stomach and transverse colon. Based on the “seed and soil” theory, the omental microenvironment provides congenial conditions for the metastatic OC cells. Interestingly, omental milky spots, specific vascularized immune cell structures containing immune cells, are significant for OC omental metastasis [57]. The cell types of both the liquid and solid TME are mostly similar while the proportion of each cell type is a discrepancy. Consistent with the large heterogeneity among OC in different patients, the composition of both tumor cells and non-malignant cells within TME including ascites, primary and metastatic lesions is diverse [66–68]. However, the underlying mechanisms related to the strong tropism of OC dissemination associated with a complex dialogue between tumor cells and stromal cells within the TME remain unclear.

### Tumor cells

The interaction between tumor cells and stromal cells formats the pre-metastatic niche in the omentum, which may be a prevalent precondition for OC metastasis [69]. For instance, OC cells could create pre-metastatic niches of the omentum by regulating the phenotype of cancer-associated fibroblasts via TGF-β1, thereby facilitating cancer progression [70]. Tumor cells could also induce mesothelial-to-mesenchymal transition (MMT) of peritoneal mesothelial cells, contributing to a pre-metastatic niche, which promotes OC peritoneal metastasis [71]. Of note, extensive ECM deposition and modification are the hallmark of metastatic peritoneal cancer in OC, contributing to poor prognosis [72, 73]. Studies have indicated that OC cells provide optimal tumor-supportive microenvironment through regulating the ECM in the TME [74]. Besides, OC cells facilitate tumor survival and invasion through modulating a desmoplastic reaction [73, 75]. TGF-β, a vital regulator contributing to tumor metastasis process related to regulating epithelial–mesenchymal transition (EMT) [76], cancer stem cell niche formation [77] and et al., can be released from tumor cells. Studies indicated that aberrant TGF-β signal pathway correlated with poor outcomes and metastasis in patients of ovarian cancer [78].

### Mesothelial cells

Mesothelial cells are a single layer of epithelioid cells covering the abdominal organs which originate from fibroblasts of the mesoderm and are characterized by both mesenchymal and/or epithelial cells. Normal mesothelial cells resist further metastasis of tumor cells by inhibiting adhesion of tumor cells to the peritoneum. However, once MMT occurs, mesothelial cells promote tumor cell invasion by promoting tumor cell adhesion to the peritoneum [79] and accumulation of CAFs [80]. Besides, mesothelial cells have less proliferative potential and are prone to senescence. Senescent mesothelial cells help OC establish peritoneal metastases by promoting tumor cell adhesion to the peritoneum [81].

### Fibroblasts

Fibroblasts and the secreted structural proteins including collagen, fibronectin, elastin, vitronectin, etc. comprise the intraperitoneal sub-mesothelial matrix. Fibroblasts are the main regulator of ECM in physiological situations [74]. Transformation of peritoneal fibroblasts into cancer-associated fibroblasts (CAFs) is one of the important causes of peritoneal metastasis of ovarian cancer. Currently, the source of intra-abdominal CAFs is not clear. The traditional belief is that peritoneal CAFs are derived from peritoneal resident cells, that is, the transformation of peritoneal resident cells into CAFs upon stimulation by tumor cells [82] while the new theory proposes that the CAFs are originated from mesothelial cells, that is, mesothelial cells undergo MMT with spindle changes, decreased expression of epithelial markers such as E-cadherin and increased expression of mesenchymal markers such as α-smooth muscle actin(αSMA) [83, 84]. Cytokines including IL-6, TGF-β could drive tumor-associated CAF phenotype and promote tumor metastasis [85, 86]. CAFs facilitate ovarian cancer progression through direct or indirect effects. As previously described, ECM deposition including collagen crosslinking plays a significant role in OC progression [73]. Studies demonstrate that CAFs assist OC progression through regulating ECM and a desmoplastic reaction [74]. Besides, CAFs secrete cytokines and promote peritoneal metastasis of OC [87, 88]. For example, fibroblasts could secrete TGF-β2, which can promote levels of CXCL12, IL-6 and VEGF-A, inducing immune evasion of cancer cells and angiogenesis [89]. TGF-β signal pathway associated molecules such as versican (VCAN) could also promote CAFs phenotypic transformation by activating the TGF-β pathway, resulting in tumor metastasis [90]. In addition, CAFs promote peritoneal metastasis by metabolic reprogramming of tumor cells. For instance, the nutrient-deficient hypoxic peritoneal microenvironment activates mitophagy and autophagy of CAFs, which provides substrates such as lactate for mitochondrial oxidative phosphorylation metabolic pathways in adjacent tumor cells, contributing to OC progression [91]. Moreover, CAFs secrete VEGF and promote tumor angiogenesis, contributing tumor progression [92].

### Adipocytes

Recent studies have demonstrated that adipocytes from peritoneal metastases provide rich nutrition for the seeding of tumor cells [93]. Thus, adipose tissue promotes tumor progression. The fatty acid receptor CD36 is highly expressed in peritoneal metastatic foci of ovarian cancer patients compared with orthotopic tumor sites. Moreover, adipocytes promote fatty acid uptake and energy metabolism in ovarian cancer cells by upregulating CD36 expression on the surface of ovarian cancer cells, resulting in peritoneal metastasis of the tumor [94]. Cysteine-rich acidic secretory proteins block interaction between adipocytes and tumor cells through inhibiting adipocyte differentiation, which alleviate OC peritoneal metastasis [95]. In addition, adipose-derived stem cells from OC patients enhance the adhesion and invasion ability of tumor cells through remodeling ECM by upregulating metastasis associated proteins such as MMPs, endothelial-specific molecule 1 [96]. Hence, adipocytes play an important role in tumor peritoneal metastasis, and elucidating the role of adipocytes on OC peritoneal metastasis is vital to improve the diagnosis and treatment of the disease.

### Neutrophils

Neutrophils belong to the myeloid lineage. The neutrophil to lymphocyte ratio (NLR) is a measure of systemic inflammation and in ovarian cancer, preoperative high NLR (> 3) serves as a predictive factor for poor survival [97, 98]. Mechanistically, neutrophils within milky spots facilitate the formation of a pre metastatic omental niches, thereby facilitating ovarian cancer cell implantation and colonization of the omentum [69]. Currently, the origin of neutrophils and their role in peritoneal metastasis of ovarian cancer are unclear. In other tumors, TGF-β signaling activation promotes neutrophils infiltration within the tumor microenvironment and supports metastasis [99]. For example, triple-negative breast cancer cells could recruit neutrophils to the local microenvironment by secreting TGF-β, involving in tumor progression [100].

### Macrophages

Macrophages participate in inflammation and immune response under physiological and pathological conditions, which are present in the peritoneal cavity. Intraperitoneal macrophages originate from 2 sources. One type is osmotic macrophages formed by the recruitment of monocytes originating from the bone marrow. The other type is tissue-colonizing macrophages formed during embryonic development. Most macrophages infiltrated in milky spots, the most frequently metastasized omental sites in OC, originate from tissue-resident macrophages [10]. However, the proportion of tumor-associated macrophages from different sources in the abdominal cavity and their function need to be further explored. Macrophages are highly plastic in response to TME. Recently, it is proposed that macrophages are classified into M1 (classically activated type) and M2 (alternatively activated type) compared with the peritoneum of patients with benign diseases, the proportion of M2 macrophage infiltration is significantly increased in peritoneal metastases of OC patients [101, 102]. Tumor-associated macrophages (TAMs) promotes OC metastatic spread through facilitating the formation of pre-metastatic niches [10]. Besides, TAMs lead to aggressive phenotype of OC cells through facilitating spheroid formation, which is associated with OC transcoelomic metastasis [32, 102]. TAMs promote anoikis of OC cells through releasing the related soluble factors, contributing the growth and peritoneal metastasis in OC [103, 104]. TAMs facilitate angiogenesis, supporting peritoneal metastasis of OC. For instance, TAMs induce angiogenesis in TME through modulating endothelial cells via affecting angiogenic pathway [105]. TAMs enhance OC metastasis by impairing T cell function [106].

### T cells

As the significant cellular types in the adaptive immune system, T lymphocytes are significant in the process of eliminating tumor cells from the host immune system. There are three main cellular subtypes of T cells in OC TME, referring to CD8+ effector cells, CD4+ helper cells and Treg cells [107]. A high CD8/CD4 ratio in TME correlates with improved outcome in OC [108], while higher infiltration of Tregs is related to worse outcome [109] due to impairing antitumor response. Tregs could release TGFβ, leading to format the tumor-promoting microenvironment and EMT of tumor cells [110]. Moreover, VEGF secreted from ascites derived T cells of ovarian cancer patients could activated VEGFR-2, which conversely inhibited T cell function [111]. Targeting anti-VEGF could augment anticancer function of CD8+ T cells [112].

### Endothelial cells

After tumor metastasis to the corresponding site, new blood vessels need to be formed at this site to supply nutrients for tumor cell survival and metastasis at the site of metastasis. Cells within the TME including macrophages, tumor cells, and mesothelial cells recruit peritoneal endothelial cells to the vicinity of peritoneal metastases and form tubular structures by secreting chemokines, TGF-β, and IL-6 to provide nutrients for OC progression [113, 114].

### Mesenchymal stem cells

At present, there are few studies have reported the role of mesenchymal stem cells (MSCs) in OC peritoneal metastasis. Resident omental MSCs acquire a CAF-like phenotype in reaction to TGF-β1 released from tumor cells [115], increasing OC metastasis through the creation of pre-metastatic niches. In addition, ovarian cancer ascites derived primary stem cells promote tumor progression by interacting with the cancer cells and macrophages to produce pro-angiogenic factors such as vascular endothelial growth factor (VEGF) and IL6 [116]. Moreover, intraperitoneal stem cells induce immunosuppressive effects through inhibiting T cell proliferation and function [117].

## The role of peritoneal fluids in OC peritoneal metastasis

Ascites is not only the result of peritoneal metastasis of tumors, but also takes the role of “porter” in implantable metastasis of ovarian cancer and provides a medium for the metastatic growth of tumors. The malignant ascites, a highly pro-metastatic environment, provides a supportive milieu for OC metastasis and thriving.

EOC metastasis is usually confined to the peritoneal cavity and distant metastasis is less frequent [11]. Malignant ascites, a clinical hallmark of poor prognosis in OC, promotes intraperitoneal transcoelomic dissemination. More than 90% of OC patients present ascites accumulation within the peritoneal cavity at diagnosis [118]. Studies indicate that the occurrence of ascites closely correlates with disease progression [29, 119, 120]. Thus, ascites microenvironment plays an important role in tumor progression.

Unlike the tumor microenvironment of solid tumors, malignant ascites presents a microenvironment of neutral PH [121], relatively mild hypoxia [122], low glucose [48], and high levels of free fatty acids [123]. It is widely accepted that the OC cells relative passively detached from the primary tumor surface could be carried to the colonization lesions through ascitic fluids [124]. Thus, except for tumor cells, the unique microenvironment within the malignant ascites contains a variety of benign cell types including mesothelial cells, fibroblasts, immune effector cells, endothelial cells, as well as a plethora of soluble factors including cytokines, chemokines, growth factors and, matrix-degrading enzymes [29, 125], supporting OC development and metastasis. Currently, studies have demonstrated that the malignant ascites microenvironment promotes peritoneal metastasis of OC by the following mechanisms.

Arterial vasodilation and venous obstruction are physiological mechanisms for ascites formation [126]. OC tumor cells cause blockage of lymphatic drainage and formation of ascites by increasing vascular permeability and/or lymphatic obstruction in the abdominal cavity [127]. In addition, stromal cells within the TME also result in ascites accumulation of OC. For example, macrophages facilitate malignant ascites development through modulating vascular permeability [128].

## Mechanism of ascites promoting OC progression

### The formation of an immunosuppressive microenvironment

Most of the immune effector cells in ascites present an immunosuppressive phenotype. Studies demonstrate that malignant ascites induces an immune-suppressed phenotype of immune cells through cytokines and metabolites [129], leading to tumor metastasis via metabolic reprogramming. Clinically, high CD4+/CD8 tumor-infiltrating T lymphocytes ratio in ascites have been related to poor outcome [108]. Moreover, compared to the primary sites and peritoneal metastases, increased CD4+ T cells has been revealed in the ascites of OC patients [108, 130]. Tregs inhibit anti-tumor immune responses, thereby the accumulation of Tregs in OC ascites is associated with advanced stage [131]. γδ T cells are an unconventional subset of T cells, involving in adaptive and innate immunity. In the clinic, compared to the primary or metastatic lesions, the proportion of γδ T cells increases preferentially in the OC malignant ascites [130]. In situations of the ascites, γδ T cells impair the normal immune response of CD8+ T cells [130, 132]. Natural killer T (NKT) cells are another peculiar subset of T cells, which are characterized by both innate NK cells and adaptive conventional T cells. Studies demonstrate that ganglioside GD3, which is overexpressed in the ascites of advanced OC patients, inhibits activation of NKT cells, leading to tumor immune evasion [133]. However, the role of γδT and NKT cells in OC metastasis remains unclear. The levels of IL6, IL10 and macrophage colony stimulating factor1 (CSF-1) are abundant in OC malignant ascites, which induce polarization of tumor-associated macrophages [134]. In OC malignant ascites, the proportions of myeloid-derived suppressor cells (MDSCs) are abundant, which correlate with poor prognosis and advanced disease stage [135]. TME induces immune-suppressive functions of NK cells. For example, NK cells existing in the OC ascites show reduced NKp30 receptor activation [136].

### Promotion of malignant biological behavior in tumor cells

The proliferation and migration of OC cells could be promoted by Lysophosphatidic acid (LPA) [137], a growth factor which is overexpressed in OC ascitic fluids. Exosomes are phospholipid-containing bilayer-enclosed extracellular vesicles secreted by cells, existing in OC ascites. They facilities OC peritoneal dissemination through mediating cell–cell communication. Ascites-derived exosomes could promote formation of the premetastatic niches within the peritoneal cavity or/and epithelial–mesenchymal transition (EMT) of tumor cells [138], playing a significant role in OC progression.

### Establishment of angiogenesis

Tumor cells and stromal cells such as CD163+ macrophages in ascites can release VEGF, TGFβ, IL-6, IL-8 [139], promoting neovascularization and supporting OC metastasis. In ovarian cancer, blocking TGF-β downregulates VEGF expression and reduces ascites formation [140]. High levels of VEGF, TGFβ, and IL-6 in ascites are inversely correlated with progression-free survival of the disease [141, 142]. Anti-VEGF therapies such as bevacizumab reduce ascites, suggesting that VEGF plays an important role in OC ascites accumulation [103].

## Conclusion

Ovarian cancer is a heterogeneous and complex disease with insidious onset, lack of effective early screening methods, and most patients are in the advanced stage at the time of presentation. The peritoneum is the most common site of metastasis of ovarian cancer, which closely corelates with the poor prognosis in patients. According to seed-pedology, the interaction between tumor cells and the peritoneal microenvironment plays an important role in the peritoneal metastasis of ovarian cancer. Ovarian cancer cells engineer metastatic sites into a soil suitable for their own survival and metastasis by remodeling the extracellular matrix within the tumor microenvironment or inducing stromal cells to undergo tumor-promoting phenotypic transformation. In addition, stromal cells promote the dissemination and growth of ovarian cancer cells in the peritoneal cavity by promoting neovascularization, helping tumor cells to immune escape, and promoting tumor cell invasion.

## Data Availability

Not applicable.

## Abbreviations

OC: Ovarian cancer
EOC: Epithelial ovarian cancer
TME: Tumor microenvironment
EMT: Epithelial-to-mesenchymal transition
YAP1: Yes-associated protein 1
Treg: Regular T
CA125: Cancer antigen 125
MSLN: Mesothelin
MMP: Matrix metalloproteinases
ECM: Extracellular matrix
MMT: Mesothelial-to-mesenchymal transition
CAFs: Cancer-associated fibroblasts
αSMA: α-Smooth muscle actin
NLR: Neutrophil to lymphocyte ratio
TAMs: Tumor-associated macrophages
MSCs: Mesenchymal stem cells
VEGF: Vascular endothelial growth factor
NKT: Natural killer T
CSF-1: Macrophage colony stimulating factor1
MDSCs: Myeloid-derived suppressor cells
LPA: Lysophosphatidic acid
